# Another Chloroform Case

**Published:** 1866-09

**Authors:** 


### ANOTHER CHLOROFORM CASE.

The following we copy from a Bloomington, Ill. paper. It occured on the second of June ult. It is another strong witness against the indiscriminate use of Chloroform as an anæsthetic for the extraction of teeth. We have lost all kind of patience with those who will persist in its constant use for simple operations. We suppose every one feels like the boy who uses fire arms, he knows that a great many other boys have been shot, but never dreams that such a thing could occur to him. In this case as in all others, a jury being called, &c., goes on to state that no blame or censure is attributable to any person. Now did that jury believe that statement? Is the minds of those who administered the chloroform perfectly at rest in regard to the matter. What, go on using an agent—that has caused hundreds and perhaps thousands of deaths—for a simple operation, till death occurs while cautions and admonitions are coming up from all quarters and in all shapes; and yet nobody to blame. We don't believe it; a coroner's jury to the contrary notwithstanding. We believe the day is not far distant when legal enactment will place this matter of the indiscriminate and almost universal use of Chloroform where it ought to be under proper restriction.

A Sad Case.—Saturday forenoon, a very estimable young lady, Miss Margaret Hughes, aged twenty one years, died from the effects of inhaling chloroform. Coroner Mark Ross called a jury, and an inquest was held. The verdict avers that the deceased came to her death from the effects of inhaling chloroform administered by Dr. C. R. Parke, and goes on to state that no blame or censure is attributable to any person. We have taken pains to learn the exact facts in the case. Dr. Lee Allen gave her chloroform last Wednesday, and extracted six or eight teeth, no disagreeable effects having been noticed. On Thursday and Friday she taught school, and Saturday forenoon, at her urgent request, Dr. Parke went with her to Dr. Allen's office and administered the chloroform. The total quantity used was less than the average amount required in such cases. At first but a small quantity was given, and two or three teeth or fangs were taken out, causing a little pain. A little more was then carefully given, and before Dr. A. had finished, the patient fainted. Although everything was done, a galvanic battery tried, &c., she never roused, but quietly and easily sank to her rest. Dr. Luce was on the walk near the office, and was immediately called in, and the united testimony of all who were present, including W. E. Hughes brother of the diseased, shows that everything possible was done without avail. Dr. Parke has administered chloroform in more than a thousand cases, but never saw a patient die before. It is one of those cases which are unexplainable. No one can tell why it is that different individuals require such different quantities of chloroform to cause insensibility. In this case the quantity taken was less than a fourth of what some persons require for the same operation.

Miss Hughes had a large circle of friends, her gentle manners having won all hearts.

